# Research on the dissemination of celebrities’ opinions based on speech act theory and potential category analysis

**DOI:** 10.3389/fpsyg.2022.1041644

**Published:** 2022-11-10

**Authors:** Xinhe Li, Chunhua Ju, Ke Li, Chenyu Wang, Chuang Li, Jie Yu

**Affiliations:** ^1^Department of Modern Business Research Center, Zhejiang Gongshang University, Hangzhou, China; ^2^Zhejiang Agricultural and Rural Big Data Development Center, Hangzhou, China; ^3^School of Management Engineering and E-Business, Zhejiang Gongshang University, Hangzhou, China; ^4^School of Statistics and Mathematics, Zhejiang Gongshang University, Hangzhou, China

**Keywords:** language style, speech act theory, opinions dissemination, potential category analysis, communication emotion

## Abstract

With the increasingly prominent role of social media in the timeliness and sharing of information dissemination, more and more research has focused on how to further improve user stickiness through social media. However, there is little consideration of the impact of celebrities’ views on user behavior in social media. The main goal of this paper is to study the influence of celebrity language style on user communication and opinions dissemination. First, it analyzes the language style characteristics of celebrities’ opinions and conducts cross-influence analysis between celebrity language style characteristics and user communication characteristics. Based on speech act theory, this study studies the influence of different language styles of celebrity Microblog on users’ communication behavior and then builds a potential category analysis model to subdivide the views of celebrities. The results show that (1) Positive expression is the most common language style element combination of celebrities, and it also shows the most effective communication effect. This shows that users like to see celebrities show an active and positive side to the outside world, can analyze external things, and express their own opinions on these contents; (2) The combination of positive emotion, external attention, and analysis can produce the best communication effect; (3) The emotion of celebrities’ opinions will affect the communication emotion of users to a certain extent, and the communication of users will have the development trend of reducing positive emotions and increasing negative emotions. Therefore, positive guidance and the dissemination of positive energy are more needed on public social platforms to minimize or avoid the dissemination of negative emotions.

## Introduction

In the Chinese dictionary, “celebrities” mean “famous persons, outstanding or conspicuous persons,” usually referring to outstanding figures in a certain industry (Wang, 2017). Microblog is one of the social media platforms for Internet celebrities to express their opinions, gain attention, and shape their personal image (McCracken, 1989; Xu et al., 2021a; Bao et al., 2022b). Celebrities frequently interact with platform users when they post information and thoughts about hot events on social media platforms (Xu et al., 2021c; Bao et al., 2022a; Han et al., 2022; Ju et al., 2022a; Wang et al., 2022). Most studies at home and abroad are from the perspective of enterprises (Langley et al., 2014) and users (Etter, 2013) to study how enterprises use social platforms to attract users’ attention, increase user interaction and improve user stickiness, but few studies show the relationship between user behavior and celebrities’ “personal brands” (Jihyun and Song, 2016; Xu et al., 2021b; Guo et al., 2022; Ju et al., 2022c). Therefore, this paper focuses on the influence of the language style of celebrities’ opinions on user communication and what kind of language style elements can increase the volume and frequency of user communication of celebrities’ opinions. The more frequently users forward celebrities’ opinions, the higher the frequency, which indicates that celebrities’ opinions will be spread to a wider range of users, and the more celebrities can enhance their personal influence and social capital, as well as their appeal to businesses or brands.

Based on speech act theory (SAT) (Searle, 1972), this study identifies the different language style elements of celebrities’ opinions, how these language style elements affect users’ communication behavior, and what combination of language style elements can more effectively spread celebrities’ opinions. According to speech act theory, every discourse is regarded as “saying something is doing something” (Boerman et al., 2017). There are three kinds of speech acts: intralingual act, illocutionary act, and implication act. The intralingual act is the act of saying these words, that is, the expression of information; the Illocutionary act is the purpose of the speech, that is, the intention of communication expressed through information. Implication act is the influence of the speech on the audience, that is, the emotional perception and action of both sides of the communication generated by the information itself and the communication intention, which has nothing to do with the early intralingual behavior. Jing et al. (2020) and others believe that replying to users through a language style similar to that of users will improve users’ evaluation and communication. Among them, perceived similarity and trust generated by perceived similarity play a promoting role. Although Internet celebrities can control the content and purpose of publishing opinions, the differences in their language styles may produce different cognitive effects among users. Therefore, this paper studies the different language style elements used in celebrities’ opinions, so as to obtain research on the communication influence of celebrities’ opinions based on language style.

In order to explore these language style elements, this study uses the short text analysis method to study the relationship between the usage of functional words and emotional words in celebrities’ opinions and users’ communication behavior. Functional words include the smallest but most commonly used words in syntactic structures such as adverbs, conjunctions, prepositions, and pronouns, which only express the grammatical relationship with other words. As the “glue” connecting sentences, Feng et al. (2020) have shown that these functional words can have a strong verbal impact on the recipients of speech acts (i.e., the users in this paper). For example, the conjunction “then” embeds the speech act into the time sequence, while “because” links the argument with the argument (Stephan et al., 2013), and uses these functional words to endow the speech act with a narrative or analytical language style. Similarly, the pronouns “he/she/it” and “they” can be used to determine the expression object of the speech act (Bird et al., 2002), giving the speech act a language style that focuses on the internal or external. Finally, different emotional styles have different effects on the credibility and influence of celebrities’ opinions. Emotional words are used to give speech acts in a positive, neutral, or negative language style (Ludwig et al., 2016). The above summarizes the different influences of different language style elements on speech acts. Although the internal elements of each language style dimension are mutually exclusive, for example, a point of opinion cannot be highly narrative and analytical at the same time. However, the combination of different language style elements can exist in a point of opinion at the same time. For example, a point of opinion can contain strong analysis, pay high attention to internal things and convey negative emotions. In this paper, we will analyze the different language styles of celebrities’ opinions by studying functional words and emotional words.

At the same time, the communication quantity and communication emotions of users will be used as evaluation indicators in communication behavior (Hancock and Dunham, 2001). According to speech act theory, users will read the corresponding information and emotions when receiving celebrities’ opinions. Users may have different concerns about a view, and there may be contradictions. However, the information and emotions contained in this view are not mutually exclusive. They can be analyzed together and focus on the use of their language styles. Therefore, this paper studies how the language style element characteristics of celebrities’ opinions affect user communication behavior, so as to subdivide celebrities’ opinions, and put forward suggestions for social celebrities who need to enhance users’ communication effect and have publicity tasks, as well as enterprises or brands looking for celebrities to promote. Firstly, this paper uses a short text analysis method to analyze the characteristics of user communication behavior from the perspectives of celebrities’ language style and user communication behavior. The characteristics of celebrities’ language style include narrative and analytical style, internal and external attention style, and emotional style; The characteristics of user communication behavior include: the number of user communication and their emotions. According to the usage of functional words and emotional words, the language style elements of the three dimensions of each celebrity’s point of view are “labeled” respectively to study how celebrities use language style to enhance interaction and communication with users. Secondly, this paper focuses on the interaction between functional words and emotional words in the viewpoint, and what kind of combination of language style elements can produce better user communication behavior. Finally, this study subdivides the opinions of celebrities through potential category analysis, so as to put forward effective suggestions and countermeasures on how to select spokesmen for enterprises or brands, and what kind of language style element combinations celebrities use to enhance user communication.

## Literature review

### Language style study

The language style is defined as different language materials and methods used by people in communication according to different communication occasions, purposes, tasks, and the temperament and quality of communicators (Xu and Du, 2021). In life, people not only express their opinions through speaking content but also achieve the purpose of communication through body language, facial expression, dress, and other ways. However, when people express their opinions and communicate with others on social platforms, there are fewer ways to help them express their opinions and communicate. Therefore, the use of language style has become an important means of expression on social platforms.

The relationship between language style and specific words will have different effects on emotional expression. Robert and others (Schindler and Barbara, 2012) think that the use of emotional words and first person pronouns will have a strong influence, while Kate and others (Muir et al., 2017) think that the use of wrong grammar and meaningless idioms will have a weak influence. Similarly, language style is significantly related to user behavior. Xun et al. (Xu and Lee, 2020) took the language style feature as a dimension to study the feature differences between users and managers when replying on different platforms. Aleti et al. (2019) analyzed the influence of the language style of celebrities on the number of user communication from the perspective of narrative and analysis style, internal and external attention style.

There are great differences in the definition and classification of language style at home and abroad, and there is no fixed standard. Eysenbach (2015) classified language styles into fact type and emotion type, and Moore and Mcferran (2017) classified language styles into body type and abstract type.

(1) Narrative and Analytical Style Study

Zhang et al. (2021) believe that the quality of a viewpoint’s narrative style determines whether users will pay attention to the viewpoint, and more specific and general suggestions are more attractive to users. Analytical style includes more complex objects and concepts, and narrative style includes more descriptive words and phrases, such as adverbs and auxiliary verbs. In addition, Seo et al. (2018) found that narrative style has a positive impact on viral communication, and Ordenes et al. (2018) found that specific word combinations can produce speech injury. Huang et al. (2016) believe that consumers will generate self perceived value through Microblog content, which will affect the recognition of Microblog celebrities.

(2) Internal and External Attention Style Study

Close et al. (2011) defined personal brands as well-known or emerging figures, who are the hot focus of social network communication, including actors, stars, online celebrities, and so on. Wang and Wang (2019) believe that celebrities can master more resources by gaining the right to speak in their industry, that is, they can get more traffic and attention on the social platform, and establish a super social relationship with users to enhance their social capital. Chung and Cho (2017) defined the super social relationship as the intimate relationship between users and celebrities, which is expressed as positive interaction and communication. Deng et al. (2021) found that celebrities who show professionalism or increase self disclosure on social media will enhance users’ attention to celebrities, thus having a positive impact on the super social interaction between users and celebrities and user communication behavior. Because the relationship between celebrities and users is asymmetric, users seem to be more inclined to forward content with an external attention style. Users’ attention to external topics and dissemination of this view can also show higher social capital, making the user look knowledgeable among peers. Therefore, this paper studies internal or external attention styles through personal pronouns (Cruz et al., 2017). The internal attention style should contain more first-person singular pronouns (I, myself, etc.,), while the external attention style should contain more second-person singular pronouns (you) or first-person plural pronouns (we).

(3) Emotional Style Study

Hopkins and King (2010) used the method of emotional analysis to study how users deal with emotional shock. Positive emotion words include “happy,” “excited,” and “thrilled,” while negative emotion words include “urgent,” “tragic” and “selfish.” Tan (2018) believes that emotion, style, and rhetoric can be the additional meaning of the non-semantic content of the text. Emotion focuses on the content and style of the form. Rhetoric, based on Skopos’s theory, is all the means to make the text effective. Emotion and style with purpose become rhetoric. Araujo et al. (2015) believe that the use of words with high emotional activation (such as good and awe inspiring) will make users more satisfied. Therefore, opinions that can arouse users’ emotions are more likely to be spread. Xie et al. (2012) proposed a multi-strategy emotion analysis framework based on an analytic hierarchy structure. Compared with the rule method based on emoticons and the emotion dictionary classification method, it has a better classification effect on Microblog emotions.

### User communication study status

User communication refers to the process in which users communicate the user experience, attribute characteristics, and other information about a specific product or service to other users through informal communication (Wang et al., 2019). This study it refers to the online communication behavior of users, which is defined by Hennig-Thurau et al. (2015) as the information published by users on social platforms and presented to other users or other businesses. Xu (2021) believes that due to the social attribute of Microblog, users in social networks will have the characteristics of interest bias, content preference, selective communication, and contact. Steffes and Burgee (2009) believe that with the rapid development of the Internet and its non-contact characteristics, the scope of user communication has expanded and the influence has increased, which is a feature that offline communication does not have. Yang (2010) believes that the communication process of information on Microblog reflects initiative, pleiotropic, timeliness, and interactivity. Everyone can speak and everyone can be concerned. Nie and Si (2019) believe that user communication can enable strangers in life to share network information through the Internet. Chen et al. (2021) believe that forwarding behavior is an important way of information dissemination on social media, and the comments added by the forwarder can reflect the emotional state and cognition. Cao et al. (2021) believe that forwarding is an important behavior of users on the Microblog platform and a key mechanism for information dissemination. Therefore, analyzing whether a tweet is forwarded by users or the amount of forwarding after a period of time can help us better understand the dissemination characteristics of information.

In the field of user behavior, celebrity and user communication are regarded as one of important ways that society influences users. It is a trendy topic that many academics both domestically and internationally have investigated. As early as 2007, domestic scholars came to the same conclusion that opinion leaders are the source of communication (Wang, 2007). Subsequently, scholars at home and abroad have studied the influencing factors between celebrities and user communication. Chaudhry and Irshad (2013) believe that celebrities play an important role in the whole process of mass consumption decision-making. Jin and Phua (2014) studied the three dimensions of celebrity endorsement, including credibility, expertise, and attractiveness. They are positive predictors of users. Credibility is the most important attribute of celebrity endorsement, which can generate the most positive user communication. Veronica et al. (2020) studied the factors that affect users’ communication of celebrity endorsements. Zhang et al. (2018) simulated the influence of user opinion leaders by building a system dynamics model and concluded that user opinion leaders, social networks, and ordinary users can affect the network reputation and user behavior by influencing the influence of user opinion leaders. Summing up the above studies, it is found that celebrities and user communication are closely related, and there is an obvious positive correlation, but there is little research on the relationship between celebrities’ language style and user communication.

## Study design

### Model construction

**FIGURE 1:**
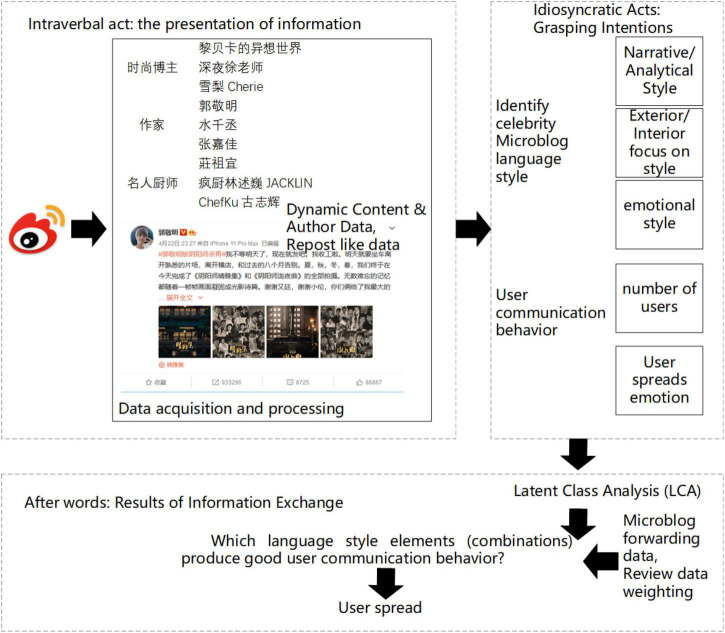
A model of celebrity opinion dissemination based on speech act theory.

According to speech act theory, information exchange between people can be summarized as information representation, comprehension intention, and emotional perception and action. Among them, the expression of information itself is regarded as an intralingual act, the communication intention expressed through the information is regarded as an illocutionary act, and the emotional perception and actions of both parties generated by the information itself and the communication intention are regarded as the post-verbal act. It also indicates the effect of information exchange between the two parties. Therefore, this study constructs a model of celebrity opinion dissemination based on speech act theory, as shown in Figure 1.

This article selects three types of celebrity Microblogs: fashion bloggers, writers, and celebrity chefs. Fashion bloggers gain user attention and traffic by posting fashion information on social platforms, which in turn gains the favor of social capital and even some businesses or brands. Writers create personal images by publishing opinions on social platforms. Choose celebrity chefs because food is one of the most discussed topics on social platforms. Although the categories of celebrities are different, the content they share is similar, so the selection of these three types of celebrities can be said to be very representative.

The article obtained a list of three types of celebrities in Microblog, and selected the top several celebrities on the list as the research object. As shown in Table 1, a total of three types of Microblog data of nine celebrities were crawled. In the case of a known celebrity Microblog ID, you can directly obtain the data of their Microblog by writing a crawler program. This study crawled a total of 7,863 pieces of data about fashion bloggers in Microblog, 8,021 pieces of data about celebrity writers, and 5,904 pieces of data about celebrity chefs, totaling 21,788 pieces; and crawled 129,214 pieces of user comments.

**TABLE 1:** Descriptive statistics of celebrity Microblog data.

Category	Celebrity	Number of Microblogs	Number of fans/10,000	Average likes	Average forwarding volume	Average comments
Fashion blogger	Rebecca’s fantasy world	2930	614	462.1	158.76	158.94
	Late at night teacher Xu	2383	1013	2410.73	1316.87	6166.79
	Sydney Cherie	2550	1083	2789.28	412.28	4156.88
Writer	Guojingming	1493	4118	7693.68	18413.31	29853.99
	Shui Qiancheng	5383	159	171.09	141.65	1218.92
	Zhang Jiajia	1145	1123	2786.85	6273.81	11315.26
Celebrity chef	Zhuangzuyi	2872	54.1	48.11	32.95	195.07
	Crazy chef Lin Shuwei Jacklin	553	106	2972.42	168.72	218.48
	Chefku guzhihui	2479	30.3	5.92	2.37	30.28

### Celebrity language style features

(1) Narrative and analytical style

Definition 1. Narrative and Analytical Style: Analytical elements include more prepositions, complex essay structures, and citations of concepts; narrative elements include more use of adverbs, conjunctions, negatives, and personal pronouns.

**FIGURE 2:**
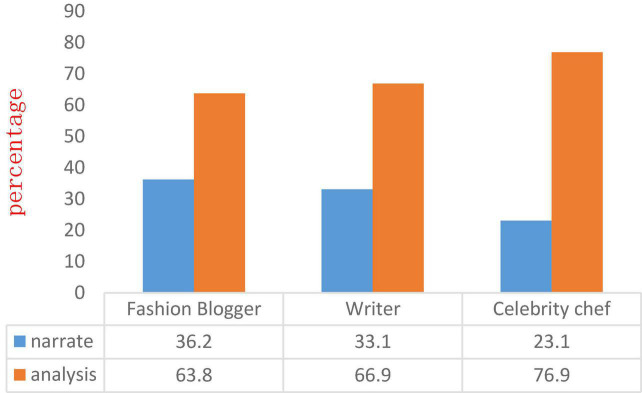
The ratio of narrative and analytical styles for each category of celebrities and all celebrities.

Determine the narrative or analysis style of each celebrity Microblog and conduct “labeling.” Finally, the ratio of narrative and analysis styles of each type of celebrity and all celebrities is shown in Figure 2.

As can be seen from Figure 2, analytical style accounts for a substantial proportion of all celebrities (63.8, 66.9, and 76.9%, respectively), indicating that celebrities tend to use more official and formal expressions when expressing their opinions on social media. Statements will be more rigorous in statement construction, which is well thought out. At the same time, there are some differences between each type of celebrity. As can be seen from Table 2, among fashion bloggers, writers, and celebrity chefs, the proportion of narrative style increases in order (36.2, 33.1, and 23.1%, respectively). The gap between fashion bloggers and writers is smaller because running personal accounts on social media has become one of the main ways for fashion bloggers and writers to promote and gain attention, fashion bloggers gain social capital through social media, and Object orientation is mostly female; writers generate buzz among users by expressing their opinions. Narrative language styles are more likely to evoke emotional perception than analytical language styles, and they will be more inclined to such propaganda methods. Although social media is also the main means of publicity for celebrity chefs, it can be seen from the crawled Microblog data that celebrity chefs’ Microblog includes three categories: cooking practices, self-brand promotion, and daily check-in greetings. Therefore, language Use a more analytical language style.

**TABLE 2:** Explicit variables are treated at different levels.

Raw data information	Explicit variable	Level	Remark
Narrative/Analytical style	U1	1	Narrative
		2	Analyze
Internal/External Concerns	U2	1	Internal
		2	External
Opinion emotional style	U3	1	Positive
		2	Neutral
		3	Negative
Number of users	U4	1	Few
		2	Less
		3	Medium
		4	More
		5	Many
User spreads emotions	U5	1	Positive
		2	Neutral
		3	Negative

(2) Internal and external attention style

Definition 2. Internal and external attention styles: Internal attention elements include more use of first-person singular pronouns (I, myself); external attention elements include more first-person plural (we), second-person singular (you), and third-person (he, they) pronouns are used.

**FIGURE 3:**
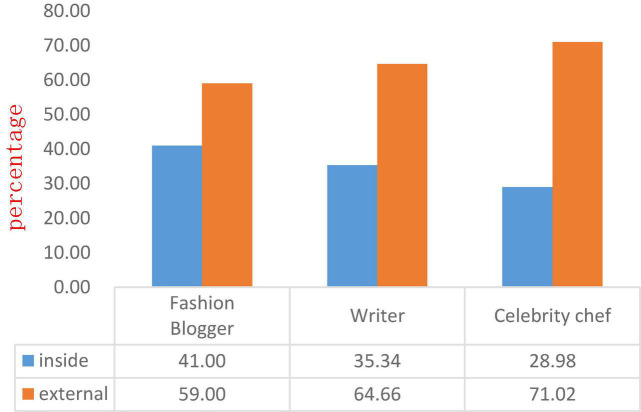
The proportion of internal and external attention styles of each type of celebrity and all celebrities.

Determine the internal or external attention language style of each celebrity Microblog, and carry out “labeling,” and finally the ratio of internal and external attention language style of each type of celebrity and all celebrities is shown in Figure 3.

As can be seen from Figure 3, the external attention style accounts for a large proportion of all celebrities (59.00, 64.66, and 71.02%, respectively), indicating that celebrities tend to pay attention to external things when they express their opinions on social media, such as Social hot topics, product promotion, etc., rarely express their feelings on social media, but there are still some differences between the three. Fashion bloggers are easy to bring their own feelings into the product introduction to resonate with users, so they get the highest internal attention score compared to the other two. It is a writer’s job to express opinions, but it is more inclined to express his attitudes related to external matters, so he obtains a high external attention score. Celebrity chefs rarely express their feelings on social media, and it is often their own feelings about the dishes that are associated with internal attention.

(3) Emotional style

Definition 3. Emotional style: Based on the emotional dictionary, the emotional value of each Microblog is calculated to determine its positive, neutral, or negative emotional tendencies.

**FIGURE 4:**
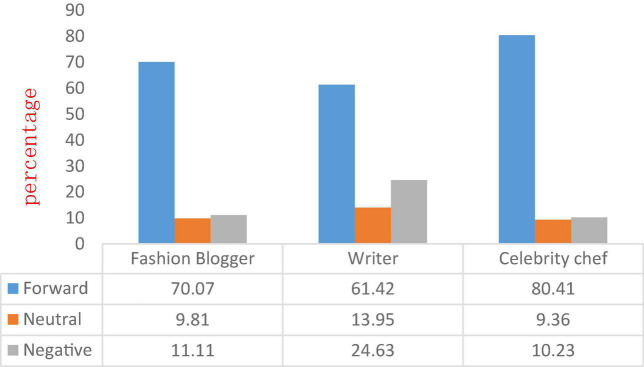
The emotional style ratio of each category of celebrities and all celebrities.

After obtaining the emotional value results of each celebrity Microblog, determining its positive, neutral, or negative emotional style, and conducting “labeling,” and finally the ratio of positive and negative emotional styles of each type of celebrity and all celebrities is shown in the Figure 4 shown.

As can be seen from Figure 4, the emotional styles of the three types of Microblog celebrities are similar. Microblog with positive emotions occupies most of the proportion (79.07, 61.42, and 80.41%, respectively), and Microblog with negative emotions accounts for the majority. A small proportion (11.11, 24.63, and 10.23%, respectively), shows that celebrities rarely vent their negative emotions in front of the public, and rarely express their non-emotional views. In addition, fashion bloggers and celebrity chefs have almost no gap in the emotional style ratio, indicating that there is uniformity in the emotional style of celebrities. It is worth noting that writers are different from celebrities in the other two categories, and the proportion of negative emotions expressed in their Microblog (24.63%) is much larger than that of fashion bloggers and celebrity chefs (11.11 and 10.23%, respectively). There may be the following reasons: on the one hand, writers usually have a more perceptual cognition of the surrounding world; on the other hand, different thinking about the surrounding world is also a way for writers to communicate with the public.

### User communication behavior characteristics

(1) The number of user spreads

The number of users retweeting is one of the most common evaluation indicators in user communication behavior research. There are increased literature on why users retweet and how to retweet. Users retweeting celebrity Microblog will achieve the effect of expanding celebrity publicity and influence. In this paper, the number of user retweets is used as a measurement index of the number of user spreads, and as an influencing factor of user spread. This paper defines the number of user transmissions as follows.

Definition 4. The number of users’ spread: The number of users’ spread is the number of retweets of each celebrity Microblog.

**FIGURE 5:**
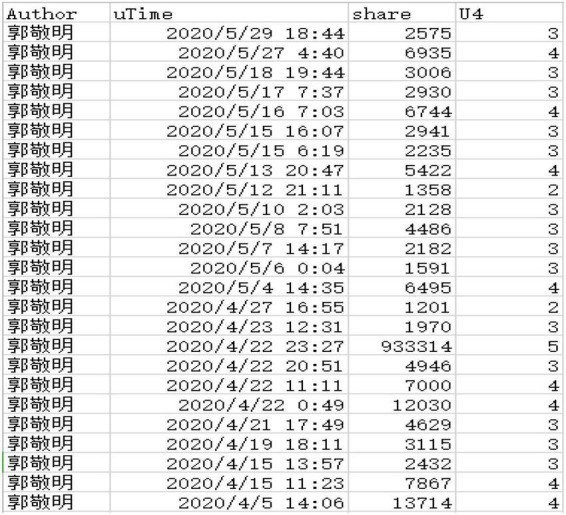
Assignment of the number of transmissions of some celebrity Microblog users.

Because the number of reposts on Microblog is too large, and there is a huge gap in the number of reposts between different celebrities. To better conduct the research of potential category analysis, the number of reposts of each celebrity Microblog is divided into five levels, 1–5, and the number of reposts of each Microblog is assigned a value, which is 1-less, 2-less, 3-moderate, 4-more, 5-more, as shown in Figure 5.

(2) User dissemination of emotions

Celebrities are opinion leaders in social platforms. Many scholars at home and abroad have studied their influence on user communication, but few studies have focused on the influence of celebrities’ emotional tendencies on user communication emotions. Therefore, this paper adds an influencing factor of user communication, that is, user communication sentiment, and defines it as follows.

Definition 5. User dissemination emotion: The user’s dissemination emotion is the overall emotional atmosphere of the comment area under each celebrity Microblog.

Like the positive, neutral, and negative emotional styles of celebrity Microblog, this study divides users’ dissemination of emotions into positive, neutral, and negative. Based on the sentiment dictionary, this paper identifies sentiment words, negative words, and degree words in celebrity Microblog comments, and comprehensively calculates the sentiment score of each Microblog to judge its dissemination sentiment classification. The weight of each emotion word *w* is set to 1, and the calculation rule between negative words and degree words is: if there are negative words, the weight; if there are double negative words, the weight *w*×(−1); if there are degree words, the weight of different degree words. The corresponding weight *w*×1.2; when the negative word is in front of the degree word, the weight *w*×0.7.


$$E_{w}=\sum_{i=1}^{n}{\left(-1\right)}^{\alpha }\cdot 1.2^{\beta }\,D\,\left(d\right)\cdot 0.7^{\gamma }\,S\,\left(e_{i}\right)$$

Among them, *e*_*i*_ represents the emotional word in the text, *S*(*e*_*i*_) represents the emotional value of *e*_*i*_, α represents whether there is a negative word in the context, when there is a negative word α = 1, otherwise α = 0; β represents a double negative word, when there is a double negative word β = 1,α = 2, otherwise β = 0, α = 0. *D*(*d*) indicates the weight of the degree word, *D*(*d*) = ∏*d*_*i*_.γ indicates the positional relationship between the negative word and the degree word, when the negative word and the degree word exist at the same time and the negative word is before the degree word γ = 1, otherwise γ = 0.

**FIGURE 6:**
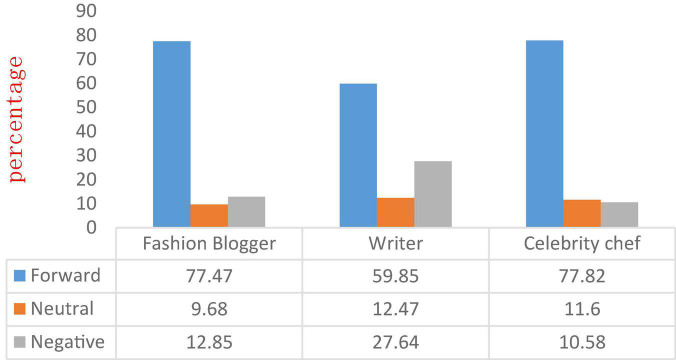
Proportion of users spreading emotions on each type of celebrity Microblog.

Due to the different number of comments on celebrity Microblog, the workload of crawling all Microblog comments is huge and unnecessary. Therefore, this paper adopts the method of crawling the 5 Microblog comments with the most likes in the comment area and judges their emotional tendencies. Like is the behavior of users agreeing with the point of view. The more likes, the more people agree with the point of view, which reflects the user’s overall emotion and atmosphere of the celebrity Microblog to a certain extent. The analysis method can be said to be representative. Through the above analysis, we finally get the proportion of users’ sentiment spread on each type of celebrity Microblog, as shown in Figure 6.

As can be seen from Figure 6, Microblog with positive emotions also accounted for the majority (77.47, 59.85, and 77.82%, respectively), and negative Microblog accounted for a small proportion (12.85, 27.68, and 10.58%, respectively), indicating that the spread of sentiment is positive. At the same time, the proportion of positive Microblog is like that of celebrity Microblog (79.07, 61.42, and 80.41%, respectively). It can be said that the emotional inclination of celebrities determines the user’s emotional inclination to some extent. However, it is worth noting that compared with the emotional characteristics of celebrity Microblog, the proportion of positive emotions decreased (respectively decreased by 1.6, 1.57, and 2.59%), and the negative emotions increased slightly (increased by 1.74, 3.05, and 0.23%, respectively), which indicates that the emotional tendencies of celebrities will affect the emotional tendencies of user communication, and user communication will tend to reduce positive emotions and increase negative emotions.

## Analytical methods

### Data preparation

The latent class analysis (Zhang et al., 2010) (LCA) is based on the latent class model (Su et al., 2021) (LCM) statistical model, and the latent class variables are used to further explain the relationship between the explicit variables of the model. Therefore, latent variables are used to estimate the relationship between explicit variables, so that the local independence of explicit variables is preserved and stabilized. Latent class analysis assumes that a small number of mutually exclusive latent class variables can be used to explain the various response probability distributions of each explicit variable, and each latent class has a specific tendency to select responses to different explicit variables (Liu and Liu, 2019). Latent class analysis stratifies a cross-categorical table of observed variables by latent unordered categorical variables, removes confounding among all variables, is conditioned on the value of the latent variable, and assumes that all dominant variables are statistically independent (Lewis and Linzer, 2011; Ju et al., 2022b). The input of LCA model data lies in the selection of explicit variables and the processing at various levels.

(1) The selection of explicit variables is of critical significance to the effect of latent category analysis. According to the research results of celebrity language style characteristics, this paper selects and determines the following five explicit variables: (1) narrative and analytical style, (2) internal and external attention style, (3) emotional style, (4) the number of user communication, and (5) user Spread emotions.
(2) Level-wise treatment of explicit variables. Due to the wide range of data such as the number of users spread among diverse types of celebrity Microblog and the large gap between the number of celebrities, not processing the crawled raw data accordingly will cause unfairness in the input data of the model and cause large errors. Therefore, all the original data were processed at various levels, and numbers were used to refer to the different levels of each explicit variable, as shown in Table 2.

Level processing instructions:

(1) Narrative/analytical style means that each celebrity Microblog is identified as having a narrative or analytical style, marked as 1 or 2, respectively; internal/external attention style means that each celebrity Microblog is identified as having internal or external attention Style, marked as 1 or 2, respectively; emotional style refers to the emotional inclination of each celebrity Microblog identified as positive, neutral or negative, marked as 1–3, respectively.
(2) Quantity of user transmission: Since the number of transmissions of each celebrity is different and the gap is large, not processing the original data will result in a large error in the model. Firstly, the number of Microblog reposts of each celebrity is processed horizontally, and the number of transmissions is defined as five levels: few, less, medium, more, and many, marked as 1–5, respectively.
(3) User dissemination of emotions: refer to the overall emotional tendencies reflected in celebrity Microblog and judge their overall emotional tendencies by crawling the 5 most liked comments on celebrity Microblog, and classify them into positive and neutral, and negative, labeled 1–3, respectively.

After the above processing, the original data is converted into numerical information, each row is celebrity data, each column is an explicit variable, and there are a total of five explicit variables, which are used as the input data of the LCA model, as shown in Table 3.

**TABLE 3:** Latent class analysis input data.

ID	U1	U2	U3	U4	U5
1	2	2	1	5	1
2	2	1	1	4	1
3	2	1	2	4	1
4	1	1	1	5	1
5	2	2	1	3	2
6	1	1	2	3	2
7	2	1	2	2	3
8	2	2	1	3	3
9	2	1	1	4	1
10	2	2	3	2	1
11	1	1	1	1	1
12	2	1	1	4	1
…..	…..	…..	…..	…..	….
21787	1	1	1	5	1
21788	1	2	1	4	1

### Model fitting and parameter estimation

(1) Model fitting

First, it is assumed that the potential categories increase sequentially from one, and then the model adaptation index obtained by the model calculation is compared and compared, so as to obtain the optimal number of potential categories, that is, how many categories are the celebrity Microblog divided into, and the result of parameter estimation is obtained.

The results of the celebrity Microblog model adaptation index are shown in Table 4. It can be seen from the table that when the number of latent categories is 3 and 4, the LMR values are less than 0.001 and greater than 0.999, respectively, indicating that there is no significant difference between the classification results of the models, and the LCA models all meet the requirements of data fitting. But when the number of latent categories is 2 and 3, respectively, the LMR values are all less than 0.001, indicating that there is a significant difference between the classification results of the models. Currently, the result of latent category 3 is obviously better than that of latent category 2. For the sake of simplifying the model, three latent categories are finally used as the optimal classification.

**TABLE 4:** Summary of fitting information for latent class analysis.

Model	k	G2/LL	AIC	BIC	ABIC	Entropy	p for LMR	p for BLRT	Class probability
1 class	10	–101565.301	203150.603	203231.259	203199.479	–	–	–	–
2 class	21	–89833.723	179709.446	179878.824	179812.087	0.999	< 0.001	< 0.001	0.2652
									0.7348
3 class	32	–85669.165	171402.330	171660.431	171558.736	0.996	< 0.001	< 0.001	0.1547
									0.7128
									0.1325
4 class	43	–85594.116	171274.232	171621.055	171484.402	0.954	> 0.999	< 0.001	0.0917
									0.1559
									0.0393
									0.7131

(2) Parameter estimation results

Parameter estimation is performed on the optimal result obtained by the LCA model, and the conditional probability of each latent category and each explicit variable is obtained. The results are shown in Table 5. It can be seen from the latent class probabilities in the table that the latent class probability of class 2 is at most 0.5128, followed by the latent class probability of class 3 is 0.3325, and finally, the probability of class 3 is 0.1547. It can be seen from the conditional probability that the characteristics of the explicit variables of each category are significantly different, and the four explicit variables of U1, U2, U3, and U5 indicate that these four explicit variables are the main influencing explicit variables of the classification.

**TABLE 5:** Model parameter estimation results.

Project				Latent class		
Raw data information	Explicit variable	Level	Label	Category 1	Category 2	Category 3
Narrative/Analysis	U1	1	Narrative	0.447	0.226	0.237
		2	Analyze	0.553	0.774	0.763
Internal/External concerns	U2	1	Internal	0.513	0.334	0.313
		2	External	0.487	0.666	0.687
Emotional style	U3	1	Positive	0.000	1.000	0.165
		2	Neutral	0.001	0.000	0.833
		3	Negative	0.999	0.000	0.001
Number of users	U4	1	Few	0.125	0.128	0.171
		2	Less	0.298	0.285	0.331
		3	Medium	0.305	0.288	0.273
		4	More	0.180	0.203	0.162
		5	Many	0.093	0.097	0.062
User spreads emotions	U5	1	Positive	0.000	0.990	0.007
		2	Neutral	0.001	0.000	0.967
		3	Negative	0.999	0.010	0.025

### Potential classification results

The potential classification of observation data is conducted according to the parameter estimation results. Taking one celebrity Microblog as an example, the selection of each explicit variable is as follows: narrative/analytical style is narrative, internal/external attention style is external attention, positive/external attention style is If the negative emotional style is positive, the number of communication is more, and the communication emotion is positive, then the corresponding explicit variable can be expressed as {1, 2, 1, 4, 1}, and it is calculated to belong to the first category, respectively. The posterior probabilities of class 2 and class 3 are 0, 0.9983, 0.0017, respectively. Therefore, this celebrity Microblog is classified as the second category. Calculate the posterior probability that each celebrity Microblog belongs to a certain category, classify all celebrity Microblog, and obtain the proportion of each category of Microblog after classification as shown in Table 6.

**TABLE 6:** The proportion of each Microblog after classification.

Project	Category 1	Category 2	Category 3
Latent class probability	15.49%	71.20%	13.31%
The actual number of Microblog in each category	3371	15531	2887
The actual proportion of each category after distribution/%	15.47%	71.28%	13.25%

It can be seen from Table 6 that there is a certain gap between the theoretical classification of the LCA model and the actual classification, because: assuming that there are Microblog with {1, 2, 1, 4, 1} explicit variable combinations, calculate them as the first, class 2, and class 3 have posterior probabilities of 0, 0.9993, 0.0007, so the probability of it being misassigned is extremely low. But if there are celebrity Microblog with the same combination, and the posterior probability of being classified as a latent category is 0.51, then the probability of the Microblog being misclassified may be 0.49 or even higher. Therefore, there is a certain error between the theoretical probability and the actual probability of the latent class.

### The segmentation features and naming of celebrity Microblog

**FIGURE 7:**
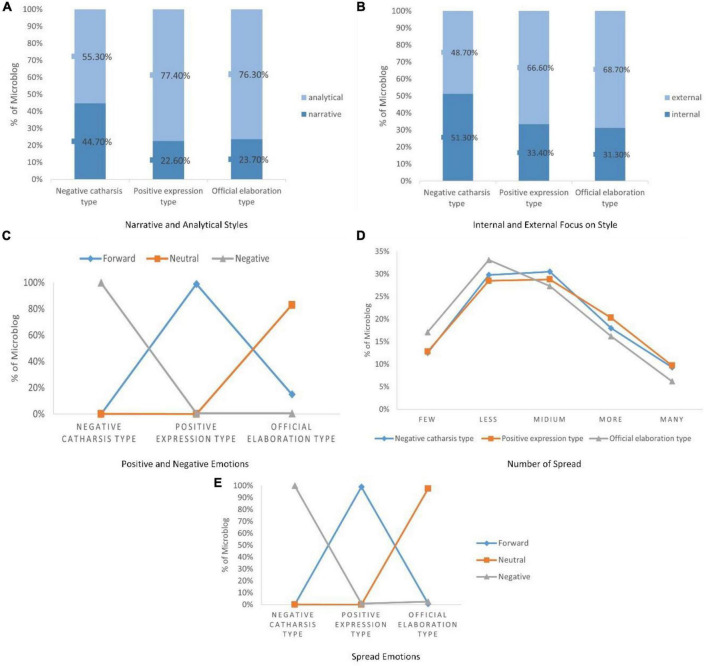
Linguistic style and user communication characteristics of celebrity Microblogs in distinct categories.

According to the parameter estimation results and potential clustering results, combined with the characteristics of the classified celebrity Microblog, the first type of celebrity Microblog can be named a negative catharsis type, the second type of celebrity Microblog can be named as internal analysis type, and the third type of celebrity Microblog can be named as external narrative type. The characteristics of various alias stream Microblog are shown in Figure 7.

(1) Negative catharsis type (15.47%)

The primary feature of this type of Microblog is a strong negative sentiment, and both celebrity’s and users’ dissemination sentiments have reached an extremely high percentage (90.9 and 99.9%). However, this type of Microblog has no special tendency in narrative and analysis style (44.7 and 55.3%), nor in the style of internal and external attention (51.3 and 48.7%). This shows that these Microblog are simply venting negative emotions, so this type of Microblog is named the “negative catharsis type.” However, from the point of view of the number of user transmissions, it has not shown a beneficial effect, and the number of transmissions is at a low level. It shows that the simple negative emotional catharsis of celebrities cannot make users resonate too much.

(2) Positive expression type (accounting for 71.28% of Microblog)

This type of Microblog accounts for the majority of celebrity Microblog and is the most important category of the model. Its primary feature is strong positive emotions, which is in sharp contrast with the first category. Whether it is the positive emotions of celebrities or the positive emotions of users, both reach extremely high percentages (100 and 99.0%). The second feature is a high degree of attention to the outside (66.6%), indicating that this type of Microblog pays more attention to external things and shows a higher social status. The third characteristic is a strong analytical style (77.4%), indicating that this type of Microblog is more formal, based on certain facts but formalized. Therefore, this type of Microblog is named the “positive expression type.” Observing the number of user communication in this category, the best communication effect was achieved among the three types of Microblog, indicating that the language style using positive emotions can drive user communication.

(3) Official elaboration type (13.25% of Microblog)

This type of Microblog occupies the smallest proportion, and its primary features are strong neutral emotions (83.3%) and a small number of positive emotions (16.5%), indicating that this type of Microblog does not show strong emotional tendencies. Like the second type of Microblog, the secondary feature is highly analytical (76.3%), indicating that this type of Microblog is more formal; the third feature is a high degree of external attention (68.7%), indicating that this type of Microblog is used in language use. show high social status. Therefore, this type of Microblog is named the “official elaboration type.” Observing the number of users’ dissemination, it can be found that the number of users’ dissemination of this type of Microblog is significantly lower than that of the other two types of Microblog, indicating that this type of Microblog has achieved the worst dissemination effect.

## Discussion and conclusion

### Discussion

Based on speech behavior theory and potential category analysis methods, this paper studies the influence of language styles from the views of three celebrities (fashion bloggers, writers, and celebrity chefs) on user communication, and studies which language style elements can produce better user communication effects. The study found that celebrities will combine different language styles to publish Microblogs, which can be divided into three categories, namely internal analysis, external narrative, and negative expression, the specific research conclusions are as follows.

Celebrity post Microblog combine different language style elements, and there are differences in diffusion influence between different Celebrities. First, the analytical type accounted for a large proportion, which illustrates celebrities prefer to use more official and formal statements when they express their opinions on social media, and the construction of the statement is more rigorous. Second, celebrities are more willing to focus on external events when they express opinions on social media, such as social hot topics, product promotion, and so on. Third, most celebrities always show a positive side, and rarely posted their opinion without emotional tendencies. The emotional styles of fashion bloggers and celebrity chefs are uniform, but the writer shows higher negative emotional tendencies.

After studying user communication behavior through potential category analysis, the following conclusions are reached. First, in different events, celebrities have different focuses and targeted use of different narrative styles. They always use the analytical type when they focus on external events and use the narrative type when they focus on internal events. Second, most celebrities show their positive attitudes when they focus on external events, but writers and chefs are more possible show negative ones when they focus on internal events. Third, user communication emotions do not have much impact in the range of diffusion, and the opinion without emotional tendencies cannot resonate with users. Four, different narrative styles or analytical styles for different celebrities will produce a noticeable diffusion effect. For example, fashion bloggers using a narrative style will produce a better diffusion effect, but writers and celebrity chefs using a narrative or analytical style do not have much impact on diffusion influence power. Five, different celebrities using internal or external styles will make different diffusion effects. Writers using an external attention style will produce a better diffusion effect, and fashion bloggers and celebrity chefs cannot produce greater influence no matter whether they use an internal or external attention style. But when writers and celebrity chefs use an internal attention style, the possibility of users’ negative diffusion would increase.

There are three types of celebrities’ opinions: positive expression type, negative cathartic type, and official elaboration type. The most common type of celebrity opinion is the positive expression type, which has higher positive emotion tendencies, strong analytical and higher external attention. Next up is the negative cathartic type, which has strong negative emotional tendencies. And the last is the official elaboration type, which has moderate emotional tendencies, strong analytical and higher external attention. Positive expression type will produce the best diffusion effect, which means users prefer to see celebrities show the positive side to the outside world, analyze external events, and disseminate such things. However, the language style which expresses a neutral mood always cannot resonate with the user, the diffusion effect of this style is the worst. Due to the strong negative emotion, the negative cathartic type stands out but still does not make a good effect on users communication. It is worth thing that narrative style does not enhance user propagation behavior from this study, although former research found that narrative content was more interesting than analytical content. Meanwhile, users are willing to forward opinions which are considered to enhance super social relationships with celebrities, that is, Microblogs that are followed by external attention. Therefore, combining positive emotion, external attention, and analytical will produce the best dissemination effect.

### Conclusion

Based on the above research findings, this paper provides some management revelations and practical guidance from the view of enterprises, consumers, and government supervision departments.

From the view of enterprises, in order to build a nice brand image, the enterprise should choose an appropriate celebrity as their spokesperson. First, it is recommended to post opinions with positive emotions, while positive emotions would promote diffusion better. Second, topics with high external attention can attract users more, such as hot events, social topics, etc., can attract users. Third, using more official and formal analytical style statements to increase user stickiness in language expression.

From the view of consumers, consumers are an important part of the dissemination of celebrities’ opinions. First, rationally look at the views of celebrities on the Internet and do not let the views of others dictate your own thoughts. Do not casually and blindly disseminate inappropriate opinions, do not blindly follow and publish statements, and create a good social media atmosphere. Second, there are many gray areas in the current online environment, and consumers should actively take responsibility for effectively reporting celebrities who spread illegal speech.

From the view of the government supervision department, the opinions and behaviors of celebrities play a key role in information dissemination. First, the emotional tendencies of celebrities affect the emotional tendencies spread by users, which makes online emotions develop in a negative direction. So, the expression of positive emotion or some public service advertisements and positive energy promotional videos should be encouraged, which could create a kind of social media atmosphere. Meanwhile, some police measures and technological means could limit the diffusion of negative emotion on public social platforms. Second, adjusting and balancing moderately the amount of information released in all kinds of fields would build a diverse sharing, relaxed, happy, and positive social media environment.

### Research limitations and prospects

This study present an advanced frontier exploration of the language style of celebrities’ opinions and influencing factors of diffusion. Due to the limitation of time, energy, and experimental environment, research till has a deep and wide space to explore, and further research can be carried out from the following aspect. First, this paper only focuses on three types of celebrities. The types this paper proposed are representative, but still, subdivide into more categories. In future research, we will compare the differences and similarities between the language style elements used by different celebrities, and which combination of language style elements can produce better effects on user communication. And celebrities’ consensus can be built based on the same points among more celebrities. Second, this study only focuses on the use of language style in text, ignoring the possible language style in pictures and videos. Future research can analyze and summarize the language style elements of celebrities reflected in pictures and videos, and combine some other social media platforms (such as Bilibili, Douyin, Kuaishou, etc.,) to conduct cross-platform language style comparisons, and get more complete conclusions. Third, this paper mainly analyzes the language style elements of celebrities’ opinions and does not study the language style existing in the user comment text and user retweet text. Future research can also analyze which language style elements users use when replying to and disseminating celebrities’ opinions and the impact of different style element combinations on user communication behaviors based on opinions’ comments and retweeted texts. As our lives become more and more socialized, the dissemination of celebrities’ opinions plays an important role. Therefore, the research on the dissemination of celebrities’ opinions has important theoretical and practical significance. In the future, based on the social network of celebrities’ fans, we can study how to adjust the relationship between celebrities’ language style and fan interaction to expand the influence of communication.

## Data availability statement

The original contributions presented in this study are included in the article/supplementary material, further inquiries can be directed to the corresponding author.

## Author contributions

CJ designed the study and conceived the manuscript. JY and KL carried out the simulation experiments. XL drafted the manuscript. CW and CL were involved in revising the manuscript. All authors have read and agreed to the published version of the manuscript.
